# Radiation Therapy in Metastatic Soft Tissue Sarcoma: From Palliation to Ablation

**DOI:** 10.3390/cancers13194775

**Published:** 2021-09-24

**Authors:** Nishant K. Shah, Nikhil Yegya-Raman, Joshua A. Jones, Jacob E. Shabason

**Affiliations:** Department of Radiation Oncology, Perelman School of Medicine, University of Pennsylvania, Philadelphia, PA 19104, USA; nishant.shah@pennmedicine.upenn.edu (N.K.S.); Nikhil.Yegya-Raman@Pennmedicine.upenn.edu (N.Y.-R.); Joshua.Jones@pennmedicine.upenn.edu (J.A.J.)

**Keywords:** radiotherapy, stereotactic radiation, soft tissue sarcoma, metastatic, quality of life, palliation, lung metastases, brain metastases, spine metastases, local recurrence

## Abstract

**Simple Summary:**

In the United States, over 13,000 patients are diagnosed with soft tissue sarcoma annually leading to over 5000 deaths per year despite aggressive treatments including radiotherapy, surgery, and chemotherapy. Although the majority of patients present with localized disease, unfortunately many will develop metastatic disease, which is generally not curable. There is growing evidence that local ablative therapies may be beneficial in patients with a variety of metastatic malignancies. In this review article, we explore the evolving role of radiotherapy in patients with metastatic soft tissue sarcoma. In particular, we review the growing role of ablative radiotherapy for oligometastatic disease, local control of the primary site, and palliation.

**Abstract:**

The management of patients with metastatic cancer is rapidly changing. Historically, radiotherapy was utilized for the treatment of localized disease or for palliation. While systemic therapy remains the mainstay of management for patients with metastatic cancer, radiotherapy is becoming increasingly important not only to palliate symptoms, but also to ablate oligometastatic or oligoprogressive disease and improve local control in the primary site. There is emerging evidence in multiple solid malignancies that patients with low volume metastatic disease that undergo local ablative therapy to metastatic sites may have improved progression free survival and potentially overall survival. In addition, there is increasing evidence that select patients with metastatic disease may benefit from aggressive treatment of the primary site. Patients with metastatic soft tissue sarcoma have a poor overall prognosis. However, there may be opportunities in patients with low volume metastatic soft tissue sarcoma to improve outcomes with local therapy including surgery, ablation, embolization, and radiation therapy. Stereotactic body radiation therapy (SBRT) offers a safe, convenient, precise, and non-invasive option for ablation of sites of metastases. In this review article, we explore the limited yet evolving role of radiotherapy to metastatic and primary sites for local control and palliation, particularly in the oligometastatic setting.

## 1. Introduction

In the United States, over 13,000 patients are diagnosed with soft tissue sarcoma annually [1]. The incidence of soft tissue sarcoma has steadily increased from 1975 to 2018 (2.2 vs. 3.4 new cases per 100,000) [2]. Even with advancements in medical treatments, there were 5350 deaths related to soft tissue sarcoma in 2020 [1]. The rate of localized, regional, and distant disease at diagnosis is 60%, 19%, and 15%, respectively. Although the 5-year overall survival is 64.7%, the majority of the mortality is driven by the stage at diagnosis [2]. The standard of care for localized soft tissue sarcoma is a combination of limb-sparing surgery and radiation therapy with or without chemotherapy. This therapeutic approach results in excellent local control [3,4]. However, survival in these patients is largely dictated by occult metastatic disease, which can be as high as 40–50% depending on the size and grade.

## 2. Current Management of Metastatic Soft Tissue Sarcoma

The standard of care for patients with metastatic soft tissue sarcoma is systemic therapy. There are a variety of chemotherapy regimens that are often tailored based on histology and the ability of the patient to tolerate such therapy. Chemotherapy is unfortunately not curative but can be effective for palliation and prolongation of patient survival. The treatment of metastatic soft tissue sarcoma was established by two landmark trials. EORTC 62012 demonstrated a progression free survival advantage (median, 7.4 months vs. 4.6 months, *p* = 0.003) of doxorubicin and ifosfamide vs. doxorubicin alone. However, there was no difference in overall survival (median, 14.3 months vs. 12.8 months, *p* = 0.076). Toxicity was significant with the combined chemotherapy group: 43% leukopenia, 42% neutropenia, 46% febrile neutropenia, and 35% anemia. Importantly, this study showed that overall response rates to chemotherapy were poor (26% for doxorubicin + ifosfamide and 14% for doxorubicin alone) [5]. The GeDDiS study showed comparable progression free survival (median, 23.3 weeks vs. 23.7 weeks) and overall survival (median, 76.3 weeks vs. 67.3 weeks) in patients treated with doxorubicin vs. gemcitabine and docetaxel. Again, overall response rates to chemotherapy remained low in each arm (19% vs. 20%) [6]. Although outside the scope of this review, numerous other agents have shown efficacy for different histologies of soft tissue sarcoma including immunotherapy.

Historically, the role of radiotherapy in metastatic soft tissue sarcoma has been limited to palliation. There is currently no established dose of radiotherapy based on soft tissue sarcoma subtypes. Classical palliative radiotherapy doses (such as 20 Gy in 5 fractions and 30 Gy in 10 fractions) have been shown to be effective at decreasing pain [7]. Given the relative radioresistant biology of sarcomas, higher doses of radiotherapy (40 Gy in 20 fractions) have resulted in more durable symptom relief [8]. Overall, the use of palliative radiotherapy remains integral in the management of patients with metastatic soft tissue sarcoma who have a poor prognosis and in whom symptom relief alone is the goal. Although, palliative radiotherapy is effective at symptom improvement, higher doses of radiotherapy may provide better durable local control and prevent progression that could cause further symptoms [9]. As such, for patients with good performance status and favorable prognosis, ablative radiotherapy may be beneficial.

## 3. Role of Local Therapy in Metastatic Cancer

The ‘existence of a clinical significant state of oligometastases’ was originally introduced by Hellman and Weichselbaum in 1995 [10]. Oligometastases is defined as a disease state with limited metastatic burden during which local ablative therapy could be curative. There is growing evidence that local control of the primary and metastatic sites in patients with limited burden of metastatic disease may offer a disease free and overall survival benefit [11]. For example, in colorectal cancer, patients who undergo resection of liver metastases can have 5-year disease free survival of 20% and overall survival of 38%. Importantly, patients with a solitary liver metastasis who undergo resection can have 5-year overall survival as high as 70% [12,13,14,15,16].

With advancements in radiation technology such as intensity modulated radiation therapy (IMRT) and stereotactic ablative body radiotherapy (SABR)/stereotactic body radiotherapy (SBRT), it has become increasingly feasible to safely deliver higher doses of radiation therapy to sites of metastases. There is no reported randomized trial investigating the role of SABR/SBRT in oligometastatic soft tissue sarcoma. This may be in part due to the rare and histologically diverse nature of soft tissue sarcoma. However, there is growing literature about the use of aggressive local and metastasis directed radiotherapy in other, more common cancer types.

Recently, two randomized phase II trials have shown promising results from local therapy in patients with metastatic non-small-cell lung cancer (NSCLC). The study by Gomez et al. enrolled patients with NSCLC who had three or fewer metastases that did not progress after initial systemic therapy [17]. Patients were randomized to local therapy (surgery or radiation therapy) to all active sites followed by standard maintenance therapy/observation or maintenance therapy/observation alone. The trial was closed early after 49 patients were enrolled due to a significant improvement in progression free survival (median, 14.2 months vs. 4.4 months, *p* = 0.022) and overall survival (median, 41.2 months vs. 17.0 months, *p* = 0.017) in the local therapy arm. Furthermore, in this study there was improvement in survival after progression with local therapy (37.6 vs. 9.4 months, *p* = 0.034). Importantly, there was no difference in grade 3 or greater toxicity with the addition of local therapy. Similarly, a study by Iyengar et al. also enrolled patients with metastatic NSCLC [18]. In this study, patients were eligible if they had up to six sites of extracranial disease including the primary with no more than three sites of metastases in the liver or lung. Patients were randomized to SABR/SBRT to all sites of disease followed by maintenance chemotherapy or maintenance chemotherapy alone. After 29 patients were enrolled, the study was closed early due to a significant improvement in progression free survival (median, 9.7 vs. 3.5 months, *p* = 0.01). Again, there was no difference in toxicity between the two groups.

Furthermore, the role of SBRT for oligometastatic disease has been evaluated in a wider variety of histologies. Specifically, SABR-COMET is a phase II study that investigated the addition of stereotactic ablative radiotherapy to standard of care treatment in patients with a wider variety of primary tumors and up to five sites of metastases [19]. The majority of patients had primary breast, colorectal, lung, or prostate cancer. Overall, there was a significant survival benefit in patients who were treated with SABR/SBRT with an improvement in 5-year overall survival of 42.3% vs. 17.7% (*p* = 0.006). There was no difference in adverse events or quality of life measures between the two groups. Furthermore, a recent economic analysis of SABR-COMET showed that SABR is a cost-effective treatment modality. Specifically, this study showed that incremental cost-effectiveness ratio was $54,564 per quality adjusted life years in the USA and $37,157 per quality adjusted life years in Canada [20]. Given these promising results, investigators have initiated a follow up phase III trial (SABR-COMET-3 Trial) that randomizes patients with oligometastatic disease to SBRT vs. standard of care with the primary endpoint of overall survival [21]. There is also now further clinical investigation testing the utility of this approach in polymetastatic cancer (>10 sites of metastases), such as the phase I trial of ablative radiation therapy to restrain everything safely treatable (ARREST) study [22].

These same principles of SBRT for the treatment of oligometastatic cancer are now being applied to patients with metastatic sarcoma with very encouraging local control results. However, there is a lack of randomized data in these patients to determine the true clinical benefit of this approach [23,24,25].

## 4. Role of Radiotherapy to Sites of Metastases

Although soft tissue sarcomas are often lumped together, different histologies often have vastly different clinical outcomes and differential responses to radiotherapy. As such there is limited evidence to help guide optimal radiotherapy doses for a given histologic subtype [26]. For the purposes of this review, we do not discuss each histology separately, but it is important to understand some nuanced differences in radiosensitivity. For example, it is well established that myxoid liposarcoma is very radiosensitive. The radiosensitivity of this histology was recently demonstrated in a prospective trial with 79 patients with localized myxoid liposarcoma of the extremity or trunk. This study showed that it was safe to de-escalate the neoadjuvant radiotherapy dose from the traditional 50 Gy in 25 fractions to 36 Gy in 18 fractions. Among 77 evaluated patients, 91% had extensive pathologic response with 100% local control [27]. Extrapolating to the metastatic setting, it may be reasonable to treat this histology with less aggressive radiotherapy regimens depending on the goals of treatment.

### 4.1. Pulmonary Metastasis

The most common site of metastases in patients with soft tissue sarcoma is the lungs. In the past, patients with metastatic disease would be treated with systemic therapy alone with or without radiotherapy for symptom palliation. Although the data are limited to retrospective, single institution data, multiple studies have shown excellent local control rates with ablative radiotherapy in patients with pulmonary metastases from soft tissue sarcoma [28].

Frakulli et al. treated 24 patients with 68 lung metastases, who were not candidates for surgical resection, from October 2010 to July 2014 with SBRT. Radiotherapy doses ranged from 30 to 60 Gy in three to eight fractions. At a median follow up of 17 months, the two-year local control was 85.9% and overall survival was 66.4%. Importantly, there were no grade 3 or greater acute or late toxicities from the intervention [29].

A study from Lindsay et al. also investigated the safety and efficacy of SBRT in patients with pulmonary metastases from soft tissue sarcoma. In this retrospective analysis, 44 patients with 117 pulmonary metastases underwent SBRT. The majority of patients (84%) received at least 50 Gy to the lung metastases. At 14.2 months median follow up, overall survival was 82% and local control was 95%. A total of 27% of patients developed new lung metastases requiring further intervention. Toxicity in the study included transient radiation pneumonitis, cough, rib fracture, chronic pain, dermatitis, and dyspnea [30].

Another single-institution study from Israel showed similar results when SBRT was used for 22 patients with 53 lung metastases. In this study, 34 lesions were less than 10 mm in size with 24 complete responses, 3 partial responses, and 7 stable disease. Eighteen lesions larger than 10 mm had slightly less favorable responses with five complete responses, eight stable diseases and five progressive diseases [31].

A study from the University of Pennsylvania examined 44 patients from 2011 to 2016 with pulmonary metastases from soft tissue sarcoma who were treated with SBRT to a median dose of 50 Gy in 4–5 fractions. In total, 82% of patients had prior chemotherapy, 66% had prior pulmonary resection, and 32% had prior thoracic radiation. The median size of the lesions was 2.0 cm. At 12 and 24 months, local control was 96% and 90%, respectively. Notably, overall survival at 12 and 24 months was 74% and 46%, respectively [32,33]. Of the 44 patients, three developed grade 2 chest wall toxicity, and one developed grade 2 pneumonitis.

Another study from the University of Rochester evaluated 52 patients with pulmonary metastases from soft tissue sarcoma from 1990 to 2006. Fifteen of those patients underwent SBRT, typically 50 Gy in five fractions, to 74 lesions (median of four lesions treated per patient). Two-year local control was 88% and 2-year overall survival was 50% [34].

Additionally, a study from Italy used a wider variety of SBRT doses including 30 Gy in one fraction, 60 Gy in three fractions, 60 Gy in eight fractions, and 48 Gy in four fractions in 28 patients with four or fewer pulmonary metastases from soft tissue sarcoma. This study demonstrated 96% 5-year local control and impressive 2-year and 5-year overall survival of 96.2% and 60.5%, respectively. Of the patients that died in the study, all had distant progression outside of the radiotherapy field [35].

Finally, a study from UCLA showed high levels of local control following SBRT (54 Gy in 3–4 fractions) to 25 pulmonary metastases in 16 patients. The most common histologies were leiomyosarcoma (28%), synovial sarcoma (20%), and osteosarcoma (16%). Local control at 43 months was 94%. There was no grade 2 or higher radiation pneumonitis or esophagitis. There was only one local progression in a patient with leiomyosarcoma who underwent 54 Gy in three fractions to a 11 by 10 mm lesion. The lesion initially decreased in size but then grew and halted in growth at 13 by 11 mm [36].

A summary of the studies assessing the role of SBRT for sarcoma lung metastases is given in Table 1.

Aside from SBRT/SABR, there are other appropriate local therapies to treat pulmonary metastases, such as metastasectomy and interventional-radiology-based ablative procedures. A systematic review of patients with pulmonary metastases from soft tissue sarcoma investigated the optimal local therapy. A total of 1306 patients with soft tissue sarcoma were evaluated where 1104 underwent pulmonary metastasectomy and 202 patients underwent SBRT/SABR. Patients who were treated with SBRT/ SABR tended to be older compared to those treated with surgery. There was a higher cumulative incidence of death in the surgery arm compared to the radiation arm (72% vs. 56%). Moreover, there was no difference in the cumulative rate of patients alive without disease between the two groups (19% for surgery and 20% for radiation) [37]. Overall, SBRT appears to be a safe and effective method of treating oligometastatic pulmonary disease, but patient selection is very important, and still prognosis and survival will be dictated by progression outside of the radiated lesion(s).

### 4.2. Spine

Spinal metastases can be very debilitating for patients. Therefore, optimal local control in spinal metastases is critical. Spinal SBRT is an important tool to give high doses of radiation to spine metastases with the hope of improving symptoms and preventing progression, which can lead to significant pain and neurologic deficits. A recently published randomized trial demonstrated the superiority of spine SBRT (12 Gy × 2) compared to conventionally fractionated palliative radiation (4 Gy × 5) in controlling pain at 3 and 6 months without increasing toxicity [38]. Durable local control with conventional palliative radiotherapy for soft tissue sarcoma is poor, which may stem from the relatively radioresistant nature of sarcoma histologies. Thus, there is rationale that local control could be improved with higher biologically equivalent radiation doses.

Although there are no randomized data, there are two retrospective single institution reviews conducted by Memorial Sloan Kettering and MD Anderson Cancer Center that demonstrate the safety and efficacy of using spine SBRT in patients with metastatic soft tissue sarcoma. Specifically, 88 patients at Memorial Sloan Kettering were treated with hypofractionated or single fraction SBRT to 120 lesions. The rates of local control were high with 12-month local failure free survival of 87.9% and overall survival of 60.6% [39]. Isolated local failures (2%) and adjacent failures (2.2%) were rare at 12 months [40]. The majority of failures were distant. Single fraction SBRT showed better local control compared to hypofractionated SBRT (90.8% vs. 84.1%). There was only 1% acute grade 3 toxicity and 4.5% late grade 3 toxicity. The group at MD Anderson showed similar results when treating 48 patients with 66 sarcoma spinal metastases between 2002 and 2013. In this study, 67% were treated with definitive SBRT and 33% were treated postoperatively. The 1-year local control and overall survival rates were 81% and 67%, respectively. There was a trend towards worse local control in patients who underwent postoperative SBRT. There was significantly improved local control with biological equivalent dose >48 Gy and if only a single vertebral body was involved [41].

Both of these studies suggest that using higher biologically equivalent doses of radiotherapy improves local control, thereby potentially reducing risk for neurological compromise and improving quality of life.

### 4.3. Brain

Similar to spinal metastases, brain metastasis progression can lead to significant neurologic morbidity. Survival after development of brain metastasis is very poor with median survival of 1.5 months [39]. Survival was longer in patients who underwent local therapy with surgery and radiotherapy [42,43]. As such, local control of brain metastases is paramount to preserving function and potentially improving survival. The role of stereotactic radiosurgery (SRS) is well established in oncology for the management of brain metastases. Multiple groups have specifically evaluated the role of SRS in the treatment of brain metastasis from soft tissue sarcoma. Recently, Sim et al. demonstrated excellent durable local control when treating 24 patients with 58 discrete lesions. The most common histology was spindle cell sarcoma or leiomyosarcoma. At 6 and 12 months, the local control was 89%. Distant brain control and overall survival remained poor at 34% and 38%, respectively, at 12 months [44]. Another study reported 88% local control in 21 patients treated to 60 intracranial metastases and median survival of 16 months [45]. Both of these studies show excellent local control with SBRT/SRS to intracranial metastases, which may preserve neurologic function compared to whole brain radiotherapy, but many patients develop distant brain metastases and have poor survival outcomes. A summary of the studies assessing the role of SBRT/SRS for sarcoma brain and spine metastases is given in Table 2.

## 5. Role of Radiation Therapy to Primary Site in Metastatic Soft Tissue Sarcoma

The literature addressing the role of local therapy to the primary site in patients with metastatic cancer is evolving. Multiple phase 3 studies have attempted to elucidate the benefits of local therapy to the primary site in different cancer types. In patients with a low burden of metastatic prostate cancer (<4 bone metastases and no visceral metastases), the STAMPEDE RT trial showed that prostate radiotherapy conferred a failure free survival benefit but no overall survival benefit [46]. In nasopharyngeal cancer, 24-month overall survival was improved with chemoradiotherapy to the primary site compared to chemotherapy alone (76.4% vs. 54.5%, *p* = 0.004) [47]. Numerous other retrospective and population-based studies have shown a survival benefit in treating the primary site with definitive radiation in patients with a variety of metastatic cancers [48,49,50,51]. However, another recently published report showed no survival benefit with adding primary tumor resection to chemotherapy (median OS: 25.9 vs. 26.7 months) in patients with colorectal cancer with asymptomatic primary tumor and synchronous unresectable metastasis [52].

Although there are no prospective trials investigating the role of local therapy in metastatic soft tissue sarcoma, multiple retrospective studies suggest a potential benefit to local therapy. Reddy et al. investigated the effects of local therapy (definitive dose radiotherapy, surgery, or surgery with perioperative radiotherapy) compared to conservative therapy (systemic therapy with or without palliative treatments). This study showed that definitive local therapy of the primary site was associated with improved median OS (17.9 vs. 10.1 months) in a population-based database [53]. Further research is needed to better define which patients with metastatic sarcoma would benefit from aggressive local therapy.

## 6. Role of SBRT in Sarcoma for Pediatrics, Adolescents, and Young Adults

Similar to adult sarcomas, the use of ablative radiotherapy in patients with pediatric, adolescent, and young adult sarcoma is evolving. In 2010, the Euro-Ewing 99 trial investigated a dose intense therapy in patients with primary disseminated multifocal Ewing sarcoma. High dose treatment included six cycles of vincristine, ifosfamide, doxorubicin, and etoposide (VIDE); one cycle of vincristine, dactinomycin, and ifosfamide (VAI); local treatment including surgery and radiation to the primary and sites of metastases; and high-dose busulfan-melphalan followed by autologous stem-cell transplantation (HDT/SCT). Secondary analysis of this study showed that patients eligible for local therapy that underwent surgery or radiotherapy to the primary and sites of metastasis had an improved event free survival at 3 years (HR = 0.7; *p* = 0.045) [54]. Given the limitations of this secondary analysis, multiple retrospective reviews have investigated the role of SBRT in this patient population.

Parasi et al. recently highlighted their institutional data on efficacy of SBRT to sites of recurrent or metastatic sarcoma in pediatric, adolescent, and young adult patients. There were 31 eligible patients with 88 lesions. The SBRT dose was 30 Gy in five fractions, and 51.6% of patients underwent radiotherapy to more than one site of disease. At 12 months, the local control rate was 83.4%. Local failure occurred in 10/57 patients with six in-field and four marginal failures. There was one late grade 3 intestinal obstruction in a patient with re-irradiation on concurrent chemotherapy. There were no acute grade 3 side effects [55].

Furthermore, a multi-institution prospective trial studied the use of SBRT in unresected, osseous metastatic sarcoma. Fourteen patients with 37 distinct treated lesions were treated to a dose of 40 Gy in five fractions. The 6-month lesion-specific control rate was excellent at 95%. In post-hoc analysis, patients who underwent consolidation of all sites had improved progression free survival (median, 9.3 months vs. 3.7 months; *p* = 0.03) and overall survival (median not reached vs. 12.7 months; *p* = 0.02) compared to patients who underwent partial consolidation [56].

The Boston Children’s Hospital group conducted a phase I/II study investigating the minimal effective dose of SBRT in pediatric patients with pulmonary metastasis from sarcoma. Dose levels included 24, 30, and 36 Gy in three fractions. A total of five patients received 30 Gy in three fractions to eight lung metastases. In that group, primary tumor histologies included Ewing sarcoma (*n* = 3), anaplastic chordoma (*n* = 1), and osteosarcoma (*n* = 1). At 6 weeks, 7/8 treated lesions had a response, but two-year local control was only 60% [57].

Given the promising data for the use of SBRT in the pediatric and adult population, there are multiple ongoing randomized trials investigating the feasibility and efficacy of SBRT to metastatic sites in patients with metastatic pediatric sarcoma. NCT01763970 is a phase II study in which pediatric and young adult patients with unresectable metastasis from sarcoma undergo SBRT to each metastatic site to a total dose of 40 Gy delivered in five fractions. The primary endpoint is lesion-specific local control at 6 months after SBRT. Additional endpoints include patient-specific local control, progression free survival, overall survival, toxicity, and quality of life [58].

## 7. Role of Combined Radiotherapy and Immunotherapy in Metastatic Soft Tissue Sarcoma

The role of combined modality treatment with radiotherapy and immunotherapy is rapidly changing in many different disease sites. There is a plethora of preclinical evidence suggesting that radiotherapy can lead to immunogenic cell death and thereby enhance the anti-tumor immune response when combined with immune modulating agents. As such, there are numerous completed and ongoing clinical trials across disease sites investigating the combination of radiation and immunotherapy [59].

A case series from Iowa investigated the use of concurrent SBRT and pembrolizumab on five patients with metastatic soft tissue sarcoma. There were three patients with undifferentiated pleomorphic sarcoma, one with intimal, and one with chondroblastic osteosarcoma. One patient developed transient grade 4 lymphopenia. There were no grade 5 toxicities. This study found that combining SBRT with PD-1 inhibitor was safe with high rates of local control (80%). Two patients in this study were felt to have enhanced local tumor regression or abscopal effect [60]. The currently open Phase II Study STEREOSARC is further investigating the role of SBRT and immunotherapy in patients with metastatic soft tissue sarcoma. The study randomizes patients to SBRT and atezolizumab versus SBRT alone. The primary objective of the study is progression free survival at 6 months [61]. Results from this trial, as well as other similar trials in other malignancies, will help elucidate the potential clinical benefit of combining radiation with immunomodulatory agents.

## 8. Conclusions

Patients with metastatic soft tissue sarcoma generally have a poor prognosis. The current standard of care is to treat patients with systemic therapy alone with or without palliative radiotherapy for symptom management. However, response rates to chemotherapy can be low, and durable symptom relief with palliative radiotherapy may be limited. With advancements in technology, there is growing evidence of the safe use of locally ablative radiotherapy to provide durable symptom relief and local control to sites of metastasis and the primary tumor. Further investigation is necessary to determine whether radiotherapy can provide a survival advantage.

## Figures and Tables

**Table 1:** SBRT for pulmonary metastasis in soft tissue sarcoma.

Study	Radiation Dose	Number of Patients	24-Month LC (%)	24-Month OS (%)
Frakulli et al., Italy [29]	30–60 Gy/3–8fx	24	85.9	66.4
Lindsay et al., Connecticut [30]	Mostly 50 Gy/10 fx, 50 Gy/5 fx	44	95	82
Baumann et al., Penn [32,33]	50 Gy/4–5 fx	44	90	46
Dhakal et al., Rochester [34]	50 Gy/5 fx	15	88	50
Navarria et al., Italy [35]	30 Gy/1 fx, 60 Gy/3 fx, 60 Gy/8 fx, 48 Gy/4 fx	28	96.2	96.2
Mehta et al., UCLA [36]	54 Gy/3 fx, 50 Gy/4 fx, 36 Gy/3 fx, 42 Gy/3 fx	16	94	NR

LC: local control, OS: overall survival, Fx: fractions, NR: not reported.

**Table 2:** SBRT for spine and brain metastasis in soft tissue sarcoma.

Study	Treated Site	Number of Patients	Number of Treated Lesions	12-Month LC (%)	12-Month OS (%)
Folkert et al., MSKCC [39]	Spine	88	120	87.9	60.6
Bishop et al., MDACC [41]	Spine	48	66	81	67
Sim et al., Moffit [44]	Brain	24	58	89	38
Flannery et al., Pittsburgh [45]	Brain	21	60	88	60

LC: local control; OS: overall survival.
